# Fracture and fracture toughness of nanopolycrystalline metals produced by severe plastic deformation

**DOI:** 10.1098/rsta.2014.0366

**Published:** 2015-03-28

**Authors:** A. Hohenwarter, R. Pippan

**Affiliations:** 1 Department of Materials Physics, Montanuniversität Leoben, Jahnstrasse 12, 8700 Leoben, Austria; 2 Erich Schmid Institute of Materials Science, Austrian Academy of Sciences, Jahnstrasse 12, 8700 Leoben, Austria

**Keywords:** severe plastic deformation, high-pressure torsion, nanocrystalline, ultrafine-grained, fracture toughness, anisotropy

## Abstract

The knowledge of the fracture of bulk metallic materials developed in the last 50 years is mostly based on materials having grain sizes, *d*, in the range of some micrometres up to several hundred micrometres regarding the possibilities of classical metallurgical methods. Nowadays, novel techniques provide access to much smaller grain sizes, where severe plastic deformation (SPD) is one of the most significant techniques. This opens the door to extend basic research in fracture mechanics to the nanocrystalline (NC) grain size regime. From the technological point of view, there is also the necessity to evaluate standard fracture mechanics data of these new materials, such as the fracture toughness, in order to allow their implementation in engineering applications. Here, an overview of recent results on the fracture behaviour of several different ultrafine-grained (*d*<1 μm) and NC (*d*<100 nm) metals and alloys covering examples of body- and face-centred cubic structures produced by SPD will be given.

## 1. Introduction

Grain refinement in metals is often regarded as a way to increase the strength without significant reduction of the ductility. In body-centred cubic (bcc) metals, the refinement has an even more pronounced effect, because grain refinement decreases the ductile to brittle transition temperature (DBTT) for standard tension experiments. It also influences fracture toughness and impact tests [1,2]. This textbook knowledge is mainly based on experiments performed in the grain size regime between a few and some several hundred micrometres. Smaller grain sizes are difficult or impossible to generate with standard metallurgical processes.

In the last 30 years, enormous efforts have been centred on the development of production techniques capable of generating ultrafine-grained (UFG) metals with grain sizes between 100 nm and 1 μm, and nanocrystalline (NC) metals with grain sizes below 100 nm [3]. In this NC regime, the strengthening effect should be even more pronounced as far as very fine grain sizes (typically smaller than 10 nm) are not regarded where softening may occur. For these materials, the uniform elongation usually decreases with some exceptions [4]. The reduction of area at failure, however, remains in many pure metals and single-phase materials relatively high. There is a vast amount of literature dealing with the ductility of UFG and NC metals, which discusses the ductility governing phenomena in this grain size regime and how to improve it (e.g. [5–7]). By contrast, the fracture toughness, which determines the sensitivity of a material to crack-like defects, has only been marginally investigated. The main reason for this circumstance lies in the fact that UFG and NC metals and alloys cannot be easily produced in large quantities with sufficient dimensions by most synthesis techniques, for example, inert gas condensation [8], pulsed electrodeposition [9] or mechanical alloying [10]. Severe plastic deformation (SPD) [11] is one exception, this method permits the production of adequate material quantities to perform fracture mechanics tests very close to the standards. This review will focus on the fracture toughness of UFG and NC materials generated by the SPD approach. The paper is organized as follows.

First some remarks to SPD and the specific microstructural features will be given. Then, fracture characteristics of fcc-metals will be presented. This will be followed by a look into the fracture behaviour of some bcc-metals. Results on an SPD-processed pearlitic steel will be presented as an example for a nanocomposite. Finally, a short summary is provided. Open questions and perspectives for this new kind of materials will be discussed briefly.

## 2. Some comments on the used synthesis process, the resulting microstructure and the fracture mechanical assessment

### (a) High-pressure torsion

**Figure 1.**
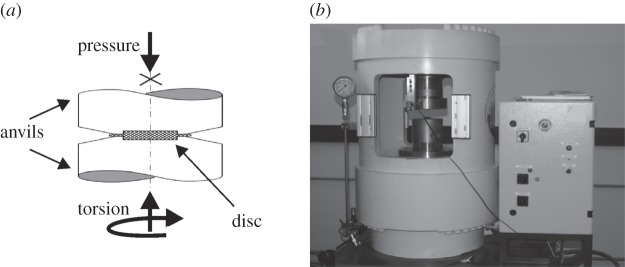
(*a*) Schematic of the HPT process, consisting of two anvils, that contains the round disc sample in between. (*b*) HPT tool with a load capacity of 4 MN (approx. 400 tons).

As the name implies, SPD it is a common term for a large variety of deformation processes, which are capable of imposing strains in the range of several hundreds to thousands of per cent on bulk metallic specimens [11,12]. Through this approach an intensive grain refinement is introduced, which is described in more detail elsewhere [13]. High-pressure torsion (HPT) is one technique, on which most of the following examples is based. The main components of this process are two anvils having a small depression in the middle. See figure 1*a*, and an example of an entire HPT facility is shown in figure 1*b*. Between the anvils round disc-shaped specimens are placed and then loaded with pressures in the range of several gigapascals. Then, the actual deformation process occurs when one anvil is rotated against the other. This rotation introduces in the samples a pure shear deformation, which is linearly dependent on the radius. Assuming that the thickness, *t*, of the sample remains unchanged during the experiment the shear strain, *γ*, can be calculated as follows
2.1
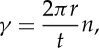

where *r* is the radius and *n* is the number of rotations. Further technical detail to this technique can be found in [14]. In comparison with some other SPD techniques, HPT has some remarkable advantages making it worth using it for the synthesis of material for basic research on the fracture behaviour of nanopolycrystals.

First, there is almost no limitation in the applicable strain without causing serious damage regarding the specimen and the tool. This is possible due to the use of high hydrostatic pressures, which prevent or delay the formation of cracks in the materials. This is also quite significant from the fracture mechanics point of view as pre-existing micro- or macro-cracks would influence the intrinsic fracture behaviour.

The chemical composition of the investigated material is only given by the pre-material. Therefore, intrinsic properties can be studied by using high purity pre-materials. Other synthesis processes, often introduce impurities, which can influence the results and make a pure grain size-dependent analysis fairly impossible. All materials presented here were produced by the same technique, namely HPT.

The HPT process is very versatile allowing the deformation of difficult to deform metals, such as tungsten or steels and process parameters, such as deformation temperature and strain can be varied in a wide range. HPT finally allows a fracture mechanics comparison of a large variety of materials produced by the same synthesis process.

### (b) Some characteristics of high-pressure torsion-microstructures

**Figure 2.**
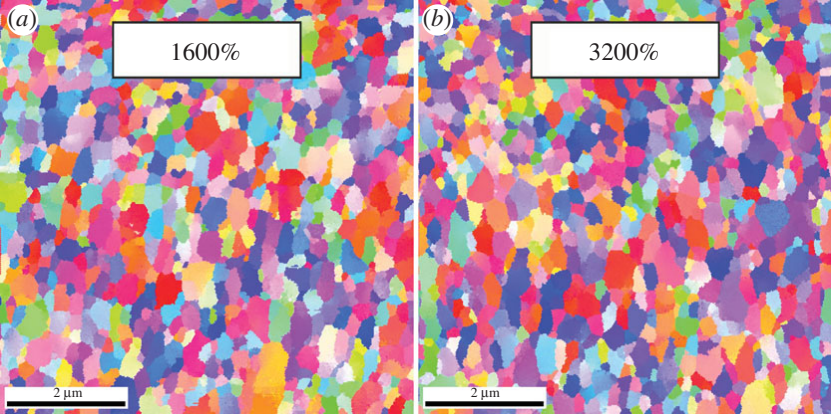
Example of an UFG Ni microstructure after strains of (*a*) 1600 and (*b*) 3200%. A cessation of grain refinement is recognizable. (Online version in colour.)

In order to understand the fracture behaviour of metals produced by HPT, it is important to mention some basic microstructural features. Since it would go far beyond the scope of this article, details of the fragmentation process are excluded here and can be found for example in [13,15]. Only one example of microstructures after very large strains is presented in figure 2. Here, the microstructure of nickel after strains of 1600% and 3200% are shown. It is evident that there is principally no difference in the grain size, even if the strain is doubled. That means that the grain size during the refinement process must stabilize and a saturation of grain fragmentation evolves indicating that an increase of strain does not change the structure any further and an equilibrium between grain fragmenting and grain coarsening processes establishes. This lower limit of grain fragmentation is mainly dependent on the purity of the material, the alloying content, deformation temperature and strain rate. So it is possible to tailor the microstructure in a wide grain size range. Finally, this saturation behaviour also means that after applying a sufficiently high number of rotations the lower limit of grain refinement can be reached at very small disc radii of the sample. So, a fairly homogeneous microstructure can be introduced in the samples even if the applied strain is inhomogeneous along the radius. The majority of results presented in this overview are determined on samples having a saturation microstructure obtained after room-temperature deformation.

**Figure 3.**
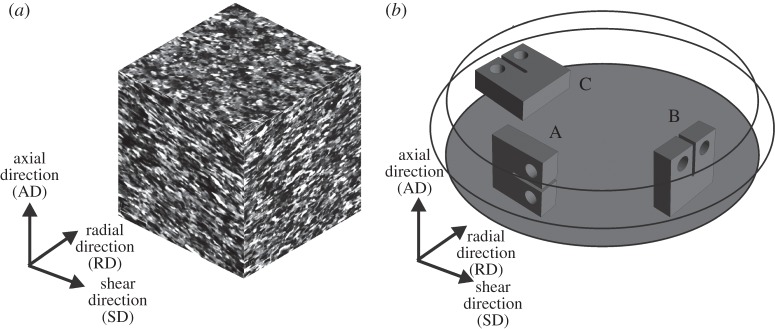
(*a*) Example of UFG iron microstructures observed parallel to the principal viewing direction. (*b*) Principal specimen manufactured from the HPT discs and their orientations with respect to the introduced coordinate system.

Another important feature of HPT microstructures is the viewing direction onto the material, which has similar characteristics typical for extremely cold-rolled structures. The common coordinate system that will be used throughout the entire paper is shown in figure 3 in conjunction with an example of saturation microstructures of HPT processed iron inspected parallel to the major viewing directions. It can be clearly seen that in the radial viewing direction (RD) the microstructure exhibits an aligned and elongated shape, which is a result of the monotonically induced shear deformation. Also in the shear direction (SD), the grains show a certain elongated shape. In the axial direction (AD), the grains appear more or less equiaxed and look somewhat larger. Very often, this structure is also termed as a pancake-structure. This distinctive alignment of the structure into the SD, which is sometimes also named tangential direction (TD), will play an important role for the fracture toughness of various presented examples.

For the characterization of the fracture behaviour, almost all experiments were performed with compact tension (CT) specimens. In order to account for the peculiarities of the microstructure, different specimen orientations were defined and the crack propagation direction and crack plane orientation coincides with the aforementioned coordinate system. For simplicity, the specimen orientation are often abbreviated with the letters A, B and C. For Orientation A, the desired crack propagation direction is into the SD and the crack plane normal is parallel to the AD. For Orientation B, the propagation direction is the AD and the crack plane normal lies parallel to the SD. Finally for Orientation C, the crack is supposed to propagate into the RD and the crack plane normal is parallel to the SD.

The typical specimen size of HPT processed materials is large compared with other synthesis processes, but at the same time small for standard fracture mechanics. Typical disc diameters used in the experiments presented had a diameter of 30 mm and a thickness of 7–8 mm. Nevertheless, it should be noted that this specimen size is still the largest one that can be successfully processed in the entire SPD community. The size of the discs leads to typical CT specimens with widths of approximately 5 mm and thickness of 2.5 mm and an initial crack length of 2.5 mm. Despite the size limitations, these specimen sizes can be sufficient to obtain useful data applying linear elastic fracture mechanics (LEFM) to measure *K*_IC_ based on the requirements and recommendations according to the ASTM standard [16]. This is possible because of the exceptionally high strength of the materials in combination with an often low fracture toughness. When LEFM was not applicable, only bound values could be measured using this approach or, as will be shown, elastic–plastic fracture mechanics (EPFM) was used to measure the critical J-Integral, *J*_IC_, again closely following the standard procedure [17]. Another very powerful tool in use was the evaluation of the crack opening displacement for crack initiation, COD_*i*_, which can also be used for calculating *K*_IC_. For that, stereo photogrammetry in the scanning electron microscope was used to generate a three-dimensional model of the fractured surfaces on both fracture halves. This allows for the reconstruction of the shape of the crack tip at the onset of crack propagation, see also [18]. Details to the specimens and the data evaluation process of the following results can be found in the individual papers.

The large differences in strength between the SPD materials and coarser-grained materials make a fracture mechanics comparison using the same specimen type and size, a very difficult task. Therefore, the results presented here are often compared with available data on coarse-grained (CG) counterparts [19–21]. To our surprise, publications dealing with pure one phase materials even for the ‘traditional’ grain size regime are rather rare.

## 3. Fracture toughness of selected severe plastic deformation materials

### (a) Severe plastic-deformed copper

Copper is maybe the most frequently studied model material in the SPD community and will be treated here first. The fracture tests were performed on CT-specimens having a width, *W*, of 20 mm, a thickness, *B*, of 10 mm and a crack length, *a*, of about 10 mm. Here only one specimen orientation was investigated, namely Orientation C (figure 3). For the fracture characterization, *J*-Integral measurements using the single specimen procedure and COD_*i*_ measurements were performed. For more detail, the reader is referred to [22]. The main focus was to perform a straight-forward comparison with a CG reference material and to clarify how fracture toughness changes when the grain size is reduced.

The typical microstructure is very similar to the example in figure 3 looking into the RD with elongated grains and a grain size of approximately 340 nm. Data on the tensile behaviour of the UFG material and the CG starting material with a grain size of about 200 μm are given in table 1. The data clearly demonstrate the impressive increase in strength through grain refinement but also the deterioration of ductility. One exception represents the reduction in area, *Z*, as a parameter for ductility, where the decrease is only weakly pronounced.

**Table 1.** Comparison of the yield strength, *σ*_*y*_, the ultimate tensile strength, *σ*_UTS_, ductility related values and the product of *K*_IC_ and of *σ*_UTS_ for UFG and CG copper. As a measure of the ductility the uniform elongation, *ε*_un_, the elongation at fracture, *ε*_fr_, and the reduction in area, *Z*, were evaluated. The product *K*_IC_⋅*σ*_UTS_ is used as an indicator for damage tolerance and for the CG-state both bound values for *K*_IC_ are incorporated.

structure	*σ*_*y*_ (MPa)	*σ*_UTS_ (MPa)	*ε*_un_ (%)	*ε*_fra_ (%)	*Z* (%)	*K*_IC_⋅*σ*_UTS_ ((MPa)^2^ m^1/2^)
UFG-Cu	443±21	601±12	3.0±0.3	18.3±1.7	75.3±4.2	∼20 000
CG-Cu	111±12	179±9	31.8±3.4	49.4±6.0	89.4±2.2	∼15 800 ÷ 49 000

**Figure 4.**
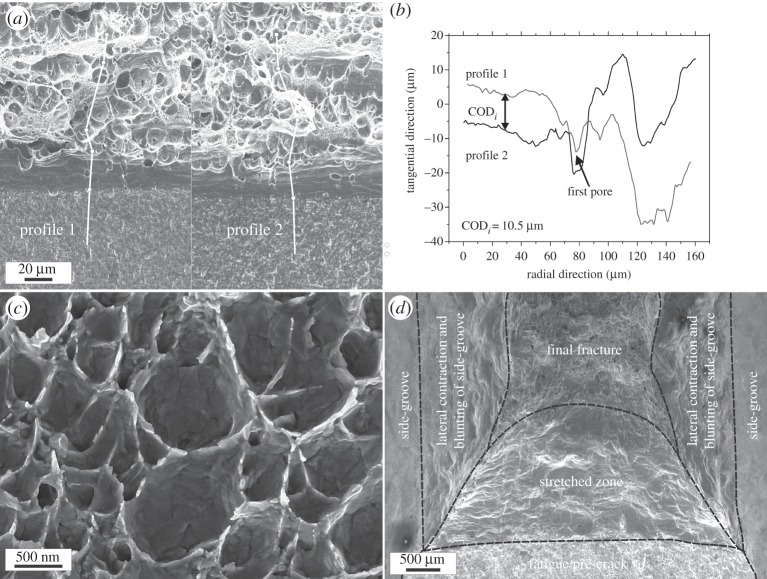
(*a*) Example of a COD_*i*_ measurement in UFG copper with fractograph taken at the identical position on both halves of the fractured specimen with a pair of corresponding crack paths taken for measuring COD_*i*_. Classical ductile dimple fracture can be observed. (*b*) Arrangement of the crack profiles extracted from the crack paths in (*a*) at the point of first crack advance. (*c*) Fine dimple structure having no obvious initiation sites. (*d*) Fractograph of CG copper with different deformation zones on the specimen. (Adapted from [22] with permission from Elsevier.)

Since measurements of COD_*i*_ are presented in this overview several times, one example describing the evaluation process should be presented in more detail. In figure 4*a*, two corresponding fractographs taken with the scanning electron microscope from the two halves of a fractured UFG-copper specimen at the same exact position are shown. Two identical crack profiles are indicated starting from the fatigue pre-crack over the stretched zone to the final fracture in the upper part of the image. These profiles are used to evaluate COD_*i*_ and the critical arrangement where the first void coalesce with the main crack. The determination of the COD_*i*_, is shown in figure 4*b*. These measurements yielded in UFG-copper in an average COD_*i*_ value of 11.4 μm.

As figure 4*a* shows, the material fails by ductile dimple fracture and even at these low magnifications dimples with typical diameters of some micrometres can be observed. These primary dimples have non-metallic inclusions as initiation sites. Their number and size is small compared with technical alloys, but they are still present even in the high purity pre-material used for this study. Besides these ‘classical dimples’, another type of dimples can be found in areas between the primary ones. These secondary dimples with a size of about twice the grain size (figure 4*c*) do not show an explicit initiation site such as nano-inclusions and will be discussed later in more detail.

Measurements of the dimple diameters investigating identical dimples on both fracture halves using stereo photogrammetry resulted in an average value of approximately 4 μm, which is much smaller than COD_*i*_. The secondary dimples are quite shallow so that their contribution to COD_*i*_ can be neglected. Therefore, a large contribution to COD_*i*_ is related to plastic deformation underneath the fracture surface. A pure relation of the dimples size to the fracture toughness could be misleading.

For comparison, in figure 4*d*, the typical fractograph of CG copper is shown and the different areas on the ductile fractured specimen are labelled. Owing to the strong lateral contraction of the specimen the COD_*i*_ could only be estimated with bound values. The upper bound value comes from adding the height of both stretched zones yielding in a COD_*i*_ of 2290 μm. This value will be very high, because it can be assumed that under plain strain conditions void initiation and coalescence would begin at lower COD_*i*_ values. A lower bound value derived from the maximum applicability of the COD concept (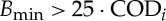 and the side-grooved specimen had a *B* of 6 mm) is given with 240 μm. These measurements clearly show that as far as the initiation toughness in terms of COD_*i*_ is concerned the fracture toughness decreases when the grain size is reduced.

**Figure 5.**
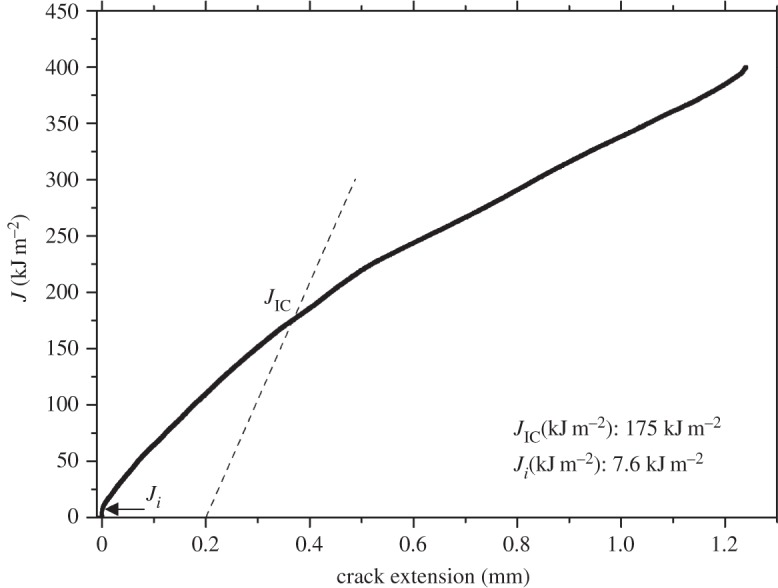
(*a*) *J*-resistance curve of UFG copper with indicated *J*_IC_ and *J*_*i*_. (Adapted from [22] with permission from Elsevier.)

The fracture resistance curve of the UFG copper (*J* versus Δ*a*) is presented in figure 5. This measurement gives a fracture toughness in terms of *J*_IC_ using the standard of 175 kJ m^−2^. For comparison the initiation toughness, *J*_*i*_, was also calculated using following relationship [23]:
2.2
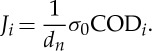

The factor *d*_*n*_, depending on stress state and strain hardening coefficient was taken to be 0.78, which is typical for a non-hardening material and characterizes the deformation behaviour of UFG materials at larger strains well. The reference stress *σ*_0_ is the average of yield and ultimate strength. *J*_*i*_ was calculated to be only 7.6 kJ m^−2^ and inserted into the *J*−Δ*a* plot in figure 5. It is clearly visible that the calculated *J*_*i*_ corresponds well to the value that would be assumed from the *J*-resistance curve in the figure. What is different to most coarse-grained ductile materials is the steep increase of *J* after crack initiation. A similar trend in the *J*-resistance curve behaviour was also observed for UFG Ti [24]. The much higher *J*_IC_ can be explained by the fact that its definition per the ASTM standard allows a stable crack advance up to 0.2 mm, whereas *J*_*i*_ defines the first physical crack advance. The further steep increase is also somewhat surprising, because in coarse-grained material the slope would normally flatten out. Another reason for the steep slope was found with fractographic observations, which showed a pronounced crack path tortuosity leading to local re-blunting processes and thus to a strong increase in the local COD with almost no crack advance. For the CG copper, a *J*-resistance curve and *J*_IC_ could not be measured because of the very low strength of the material.

For a direct comparison with other materials very often critical stress intensities are demanded. From textbook knowledge, *J*_*i*_ can be directly converted into *K*_IC_ or *K*_*i*_ with
2.3
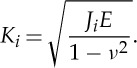

With typical values for Young's modulus, *E*, Poisson's ratio, *ν*, a fracture toughness of *K*_*i*_=33.4 MPa m^1/2^ is obtained for UFG copper. It should be noted that in CG metals the difference in *J*_*i*_ and *J*_IC_ is small; hence a calculation of *K*_IC_ from *J*_*i*_ or *J*_IC_ often give similar values. This is different for the present UFG Cu, where *K*_*i*_=33.5 MPa m^1/2^ for crack initiation and *K*_JIC_=160 MPa m^1/2^ calculated from *J*_IC_ following the ASTM standard. It is evident that *K*_*i*_ is a clear lower limit for the *K*_IC_ value, however, it remains an open question if *K*_JIC_ is a useful measure of fracture toughness for such materials.

When the results for the UFG state are compared to the CG copper in terms of stress intensities and use the lower estimate for COD_*i*_ with 240 μm and re-calculate this value into a *K*_IC_, a result of 88.5 MPa m^1/2^ is obtained. Using the upper limit of COD_*i*_ with 2290 μm, a KIC of 273 MPa m^1/2^ is calculated. In common textbooks, a value in the range of 100 MPa m^1/2^ can be found [25], which shows that the lower estimate makes sense. The initiation fracture toughness of UFG copper, derived from *J*_*i*_, which is a very conservative approach, is clearly lower with 33.4 MPa m^1/2^ and should only be considered as a lower limit. The same decrease in fracture toughness has been shown before comparing the COD values of the two material states. In other studies, dealing with UFG, nanotwinned and CG copper, the opposite, namely an increase in fracture toughness, was found when the important microstructural length scale, i.e. grain size or lamella spacing [26,27] was decreased. This can be widely explained by the LEFM assumptions that were made to evaluate an apparent fracture toughness.

Nevertheless, a single perspective into the fracture toughness is often not enough for the material performance and the combination of material parameters is important. A more informative measure for the material performance will be the damage tolerance of a material. For resisting damage, the strength is also important, as otherwise a structure may plastify before the crack sensitivity comes into play. When one defines the damage tolerance of a material as the product of strength and toughness (*σ*_UTS_⋅*K*_IC_) the material performance of the UFG material becomes more competitive (table 1).

It should be noted that the COD_*i*_ approach to calculate *K*_IC_ gives very conservative values and does not involve any subcritical crack growth that could be present within the small nonlinearity allowed by the ASTM standard. Therefore, the fracture toughness in terms of *K*_IC_ could also be significantly larger.

### (b) Severe plastic-deformed nickel

In this study, the influence of the testing direction was focused on and all three testing directions, as introduced in figure 3, were tested with *J*-Integral measurements using a multiple specimen test procedure and additional COD measurements. For the latter measurements, specimens were deformed beyond crack initiation and then cut in the mid plane, ground, polished and the resulting crack profiles were digitized and arranged to measure COD_*i*_ at the former crack tip.

**Figure 6.**
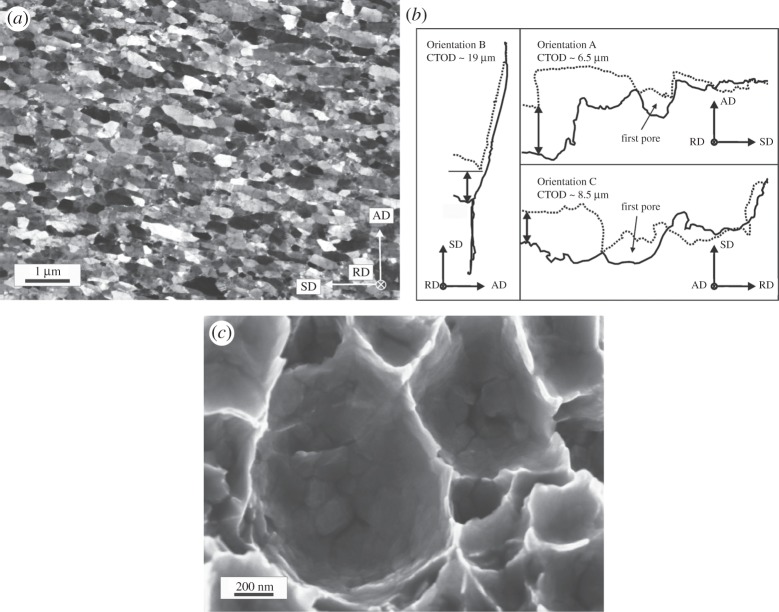
(*a*) Representation of the microstructure. (*b*) Crack profiles evaluated for the differently oriented specimens. (*c*) UFG dimples displaying individual grains in the interior. (Adapted from [28] with permission from Elsevier.)

The typical microstructure observed parallel to the RD is shown in figure 6*a* and consists of elongated grains with an alignment almost parallel to the SD with a grain size of about 300 nm. The ultimate strength can be found in table 2 and more details to the following results can be found in the corresponding paper [28].

**Table 2.** Overview on the Ni results.

material state	*J*_IC_ (kJ m^−2^)	*K*_JIC_ (MPa m^1/2^)	*σ*_UTS_ (MPa)	*K*_JIC_⋅*σ*_UTS_ ((MPa)^2^m^1/2^)	*K*_IC_ (from [COD_*i*_) (MPa m^1/2^)
Ni-SPD-OA	96.5	145.6	1400	∼203 000	60
Ni-SPD-OC	99.8	148.1	1400	∼207 000	69
Ni - 95 μm	225	222.4	341	∼76 000	—

The COD results are presented in figure 6*b* with the crack profiles and in table 2. The interesting fact is that for Orientation B the crack exhibits a crack deflection perpendicular to the desired crack propagation direction and COD_*i*_ in this orientation shows a much higher value as for the other directions. It should be noted that due to the deflection and the connected shearing of the crack flanks, the reconstruction to the point of the first large visible crack advance and not of the coalescence with the first void was accessible. The new crack propagation direction is very close to the one of a specimen with Orientation A into the SD. The fracture resistance into the desired crack propagation (AD) seems to be higher than the one in the SD and fracture toughness of Orientation A can be regarded as the lower limit of fracture toughness for Orientation B. The reason for the crack deflection and the anisotropy in the fracture behaviour is found in the typical microstructure, which exhibits an alignment of the grains into the SD serving as the weaker crack path with lower crack growth resistance. Moreover, the typical shear texture found in HPT processed materials might play a role.

The fracture morphology has for all testing directions a ductile appearance consisting of larger primary dimples with non-metallic inclusions as initiation sites, which are interconnected by secondary dimples consisting of several grains (figure 6*c*). A similar fractography was also found for copper. Only for Orientation B, the morphology is somewhat different with elongated features due to the shearing of the crack flanks under mixed-mode loading conditions.

Experimental studies on NC Ni investigated by transmission electron microscopy observed the formation process of such voids, but with a much smaller grain size [29], and these observations can also be used to explain the formation process here. Void initiation takes place at grain boundaries and triple junctions forming nanovoids. Such voids could also be formed here in the same way and then coalesce along the grain boundaries forming larger voids. Through the coalescence of several of such voids the final secondary voids may be formed. This would also lead to the same observation as here in figure 4*c*, where within these small secondary dimples individual grains can be discriminated. Since in these experiments, individual grains are visible with their grain boundary surfaces it could be concluded that locally an intercrystalline fracture type occurs leading to the dimple formation, which is normally associated with a brittle behaviour. An intercrystalline crack path has also been found before with molecular dynamics (MD) simulations [30,31]. In these simulations performed with much smaller grain sizes, nanovoid formation ahead of the crack tip was found. These nanovoids or nanocracks connect with the crack tip leading to the intercrystalline crack path as seen here within the secondary voids. Although these simulations postulate plastic deformation with some dislocation activity around the crack tip and the formation of twins, the calculated fracture toughness would be very low which might be also a consequence of the much smaller grain sizes that need has been used in the simulations.

For a grain size comparison with available literature, data on CG nickel *J*-measurements were performed which showed for Orientation A and C similar values of *J*_IC_ with 96.5 and 99.8 kJ m^−2^, respectively (table 2). Somewhat lower values were found by using the stretched zone width to calculate *J*_IC_. For Orientation B, no Mode-I propagation occurred and the principle of measurement was not applicable. Compared to a coarse-grained material with a grain size of approximately 95 μm [19] (table 2), there is again a clear reduction in fracture toughness. When we make a similar comparison as done for copper by looking again onto the product^1^ of (*K*_JIC_⋅*σ*_UTS_) as an indicator for damage tolerance one can see even an improvement due to the high strength of the UFG material. A comparison between UFG-copper and UFG-nickel, considering in both cases *K*_IC_ inferred from COD_*i*_, reveals that the fracture toughness is higher in UFG-Ni.

### (c) Severe plastic-deformed iron

The typical microstructure in SPD iron is very similar to the one presented for Ni with an average grain size of about 200 nm consisting again of elongated and aligned grains close to the TD looking into the RD. The ultimate strength of the material is extremely high with about 1.6 GPa. In a tensile test, failure occurs through shear band formation and propagation and due to this very localized deformation mechanism the ductility is extremely low. Further details to the results can be found in [32].

**Figure 7.**
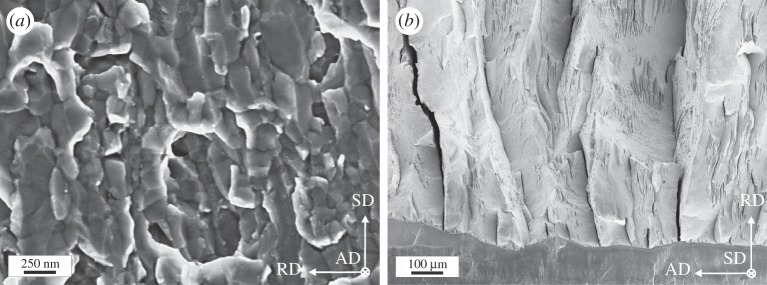
Characteristic fractography features of SPD iron. (*a*) Typical fracture surface for Orientation A with intercrystalline fracture. (*b*) Fractograph of Orientation C with delaminations.

In Orientation A (table 3), the fracture toughness measurements show values of about 14 MPa m^1/2^. This remarkable low fracture toughness can be connected to the typical fracture surface exhibiting intercrystalline fracture with only limited signs of plastic deformation (figure 7*a*). One reason for the intercrystalline fracture seem to be the aligned microstructure serving as a weak crack path. Despite the low value, the failure type contains also a certain amount of plasticity, because the calculated COD is much larger than the grain size, see also [32].

**Table 3.** Overview on the results on iron and comparison with CG material.

material state	*K*_IC_ (MPa m^1/2^)	*σ*_UTS_ (MPa)	*K*_IC_⋅*σ*_UTS_ ((MPa)^2^ m^1/2^)
Fe-SPD-OA	14.2	1616	∼23 000
Fe-SPD-OB	36.2	1616	∼58 500
Fe-SPD-OC	49	1616	∼79 200
Fe-38 μm	202	299	∼60 400

In Orientation B, very similar to Ni, a crack deflection almost perpendicular to the expected direction was observed. This direction (SD) is the same as the crack propagation direction for samples with Orientation A. The reason for the deflection can be found in the low fracture toughness found for crack propagation along or close to SD (orientation A). For Mode-I propagation, the crack would have to cleave the elongated grains or it would have to take a more tortuous crack path by passing the elongated grains in an intercrystalline manner. Both scenarios seem to have a higher fracture resistance than the one along the SD (table 3). Since the crack runs again close to the SD the failure type is again an intercrystalline one. Despite this, the fracture toughness is higher compared with Orientation A. The reason for this can be found in the fact that the crack-bifurcation reduces the local driving force for crack propagation. For a short-kinked crack, the local driving force is only about 50% of the global one [33]. Therefore, without considering any further plastification, the fracture toughness in Orientation B should be about twice as large as for Orientation A, which has to be regarded as a lower bound value since for a pure Mode-I propagation higher driving forces, i.e. fracture toughness would be needed. In the phenomenology very often used to describe the fracture behaviour of layered structures, laminates or rolled structures, this specimen orientation can be termed crack arrester configuration.

In Orientation C, a completely different fracture surface can be found. There are secondary cracks or delaminations which have the same crack plane as specimens with Orientation A, which indicates that the low fracture toughness along the SD-plane seems to trigger the formation of these delaminations. The positive effect onto the fracture toughness (table 3) is well known for example from other fracture toughness studies and very often from charpy-impact tests [34–36]. The formation of the delaminations during the loading process leads to a change in the stress state with a reduced stress triaxiality. It allows further deformation before failure resulting in a higher fracture toughness due to a delamination induced toughening effect and the orientation is often called the crack divider orientation. Experiments with small pre-loads and consecutive fatigue loading proved that the delaminations are formed spontaneously during the loading before reaching the load needed for the onset of crack propagation and that they are not intrinsically present in the as-deformed material. During the loading process, the delaminations are formed and reduce the through thickness constraint. The formation of the delaminations then leads to a combination of crack growth locally by shear fracture and a contraction of the remaining ligaments between the delaminations. This indicates that the material also exhibits a pronounced R-curve behaviour [37].

The results showed large differences in the fracture behaviour due to anisotropy. The pronounced anisotropy can be attributed to the deformation induced elongated microstructure. This microstructure causes a low fracture resistance along the elongated microstructure and in the other testing orientations crack deflection or the formation of delaminations causing an extrinsic increase in the fracture toughness. The key feature seems to be the intercrystalline crack path that will be discussed in the next section in more detail.

In view of varying the grain size, the material is compared to a coarse-grained state [20] (table 3). Especially here the considered orientation plays an important role. In general, there is a trend of decreased fracture toughness when the structural size becomes smaller. Nevertheless, the improvement in strength is exceptional through SPD processing and partly compensates for the fracture toughness decrease considering the product of strength and toughness.

### (d) Influence of testing temperature in severe plastic-deformed iron

**Figure 8.**
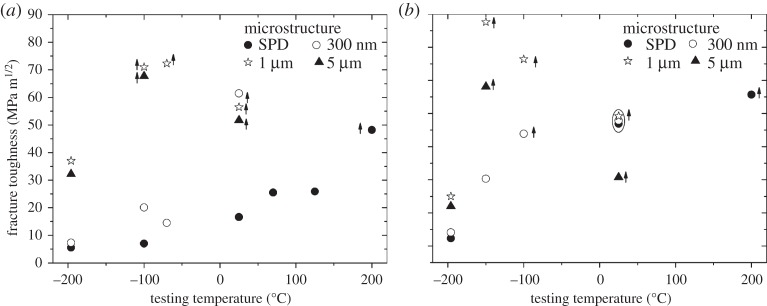
Summary of the fracture toughness measurements for Orientation A (*a*) and C (*b*). Arrows next to the data points indicate that the values are lower bound values for the critical fracture toughness.

So far the reported results were obtained in room-temperature experiments. An important aspect dealing with bcc metals is the testing temperature and the change of the ductile to DBTT with varying grain size. In this context, iron was again the focus. With subsequent well-defined heat-treatments, the grain size was varied between 200 nm and 5 μm. In addition, the influence of the testing direction was also accounted for. For details, see [38]. A summary of the results is presented in figure 8 for Orientation A and C. It should be noted that arrows next to the data points indicate that these specimens did not fulfil small-scale yielding conditions. The decrease in strength and increase in toughness with rising test temperature leads to an ‘apparent’ but not physical related drop in the measured value especially for larger grain sizes, which can then only be regarded as a lower bound value for an LEFM experiment.

In figure 8*a* for the ‘weak’ crack propagation direction (Orientation A), it is apparent that the smallest grain size (SPD) exhibits the smallest fracture toughness for the entire test regime. For the next larger grain size (300 nm) at liquid nitrogen, the toughness is comparable to SPD, but there is a huge difference at room temperature and the measurement already becomes invalid. For larger grain sizes (1 and 5 μm), the fracture toughness is about four times higher compared with the SPD state at liquid nitrogen and the values become invalid even at lower testing temperature compared with the 300 nm state. This shows very clearly that the DBTT moves to larger temperatures for grain sizes below 1 μm.

Very often it is reported that the DBTT decreases with decreasing grain size. This is often explained by comparing the effective yield stress at the crack tip with the brittle fracture stress and the resulting fracture type depends on which stress value is reached first (e.g. [39]). These considerations imply that the DBTT decreases with decreasing grain size, because the critical fracture stress increases with decreasing grain size more strongly than the effective yield stress. This concept works well for a transition from ductile fracture for the upper shelf of the transition to transcrystalline cleavage fracture for the lower shelf region. However, here in the small-grained materials (SPD and 300 nm) intercrystalline fracture is present, which changes gradually into a ductile fracture type for higher testing temperatures. For grain sizes of 1 μm tested at −196°C, a mixture of intercrystalline and transcrystalline fracture occurs and for a 5 μm specimen pure transcrystalline fracture was found exhibiting in both cases a much larger fracture toughness compared with the UFG states and for higher testing temperatures the fracture type becomes ductile as traditionally assumed for the explanation of the DBT behaviour. Putting that into perspective, it can be concluded that there is along with the temperature-dependent DBT another DBT in this grain size regime. By varying the grain size and going below a critical value of about 1 μm, there is a transition from transcrystalline to intercrystalline fracture. This intercrystalline crack path with a relatively low fracture toughness also played an important role for the strong anisotropy in the fracture behaviour discussed in the previous section.

At first, the intercrystalline crack path could be associated with classical embrittlement by sulfur or phosphor even when, as here, very pure materials were used. On the contrary larger grain sizes, for example, the 5 μm specimen, tested at −196°C do not display the intercrystalline behaviour anymore, even if this specimen should have larger amounts of segregates on the grain boundaries. This is because the fraction of grain boundaries decreases when the grain size becomes larger during heat treatment and the concentration of these segregates increases. Consequently, it can also be thought as an intrinsic behaviour of iron showing a transition of the fracture type at a grain size of approximately 1 μm. Interesting to note is that in another field of plasticity a similar fundamental change has been observed in cyclic deformation experiments where typical cell structures were not found for the UFG regime [40]. Similar observations exhibiting an intercrystalline fracture behaviour are reported in various references dealing with grain sizes typically 1 order of magnitude smaller than the one observed here, which is a suitable grain size regime for MD simulations (e.g. [41]). In this contribution, the crack can advance by coalescence with nanovoids formed ahead of the crack tip forming an intercrystalline crack path depending on the local orientation. In another theoretical contribution, the intercrystalline crack path is explained on the basis of the Cottrell model for cleavage, which was expanded to very small grains where only a limited number of dislocations fit onto the slip planes and the pile-up [42]. An important reason for the intercrystalline crack path observed here seems to be the elongated grain shape, which serves as a smooth and therefore beneficial crack path compared to an equiaxed structure. However, along with the work performed on iron, other bcc-metals have also been investigated in our group recently. For Orientation A (weak orientation in Fe), intergranular fracture behaviour was frequently found, for example in Ta, Cr and also Mo. In the case of Mo, the intercrystalline crack path has also been predicted by MD simulations [43]. Although, there are also counterexamples, for example, Nb and Va, which exhibit ductile fracture but still having an elongated grain shape. This indicates that the elongated grain shape must not be the only controlling factor for the observed intercrystalline crack path in iron. The reason for these differences in the bcc metals is an open question.

For the strong testing orientation in figure 8*b*, the tendencies are very similar, however, for the submicrocrystalline state the fracture toughness is higher compared with Orientation A. This can be again explained by the formation of delaminations in this testing orientation as shown is a previous section, which form for the SPD state and also the 300 nm specimens. Despite the significant decrease of toughness with decreasing temperature, the fracture mode remains unchanged. The formation of delaminations is also known to decrease the DBTT in the microcrystalline (MC) regime [44]. This could not be shown here, which might be connected with the investigated grain size range spanning from the micrometre to the nanometre regime, in which the strength increase is more pronounced than in the MC regime.

### (e) Nanostructured pearlitic steel

**Figure 9.**
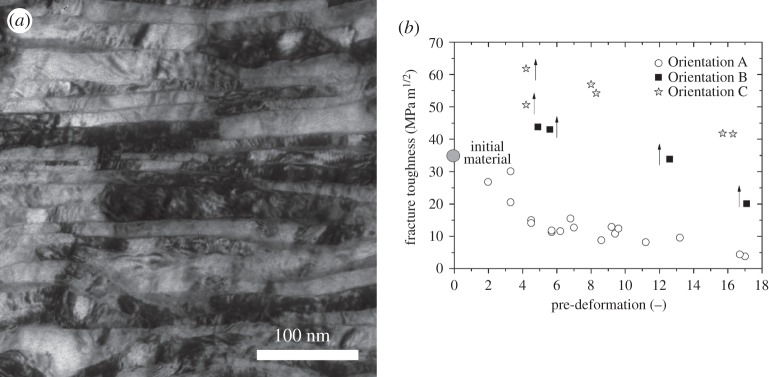
(*a*) Nanostructured steel after straining of approximately 1600% with the lamellae fully aligned into the SD. (*b*) Summary of the fracture toughness measurements.

As a last example results from a conventional pearlitic steel used for rails are presented. This study was of industrial relevance and performed in order to get a deeper insight into the formation of the so-called head-checks, which are formed on the heads of rails under certain loading conditions regarding the wheel-rail contact. The contact provokes an intense local shear deformation of the rail surface [45]. This phenomenon observed in many sliding contact problems can be systematically studied by HPT deformation of the as-received material. The fracture toughness for different microstructural states, i.e. pre-strains and specimen orientations was studied, which seems to be the key-factor for the understanding of the head-check phenomenon. The initial material has a cementite lamella spacing of about 200 nm. The induced shear deformation leads to a gradually increasing alignment of the pearlitic colonies into the SD connected with a strong reduction of the lamella spacing and a thinning of the cementite lamellae. The microstructural evolution in detail can be found in [46]. Owing to these microstructural changes, the hardness increases from about 260 HV to about 800 HV. Here, the structure after extreme high deformations (1600%) is shown in figure 9*a*. In comparison with the starting material, the structure has transformed into a nanocomposite structure with a lamella spacing of about 20 nm and a full alignment of the entire structure within the sample parallel to the SD and such structures are also present on the very top of rails. The impact of this nanostructuring process onto the fracture toughness is shown in figure 9*b* [47,48].

For Orientation A, one can see a strong decrease of the fracture toughness with increasing deformation down to values of only 4 MPam^1/2^. This deterioration is so intense that after this high pre-strain the material is comparable to inherently brittle materials, such as ceramics and is also thought to be an important factor to understand the head-check formation. The reason for the behaviour can be related to the alignment of the structure into the direction of the propagating crack. In the other testing directions, the situation is completely different. In orientation B, there is again a crack bifurcation inducing a higher globally measurable fracture toughness. Here in the extreme case after the highest pre-deformation, the alignment is perpendicular to the crack orientation. In Orientation C, again delaminations can be found having their origin in the low fracture toughness found for cracks propagating along the SD (orientation A). This result demonstrates that in the same material having an exceptionally high hardness the fracture toughness can vary by a factor of 10. In another perspective, a material suspicious to a world record regarding the combination of strength and fracture toughness (Orientation C) is tested in another direction (Orientation A) at the same time performing rather poor.

## 4. Summary and perspectives

This overview showed that in severely plastically deformed materials the fracture toughness can be smaller than in the CG counterparts. However, it was also shown that the damage tolerance in terms of the product of fracture toughness and strength can be remarkable. Additionally, in contrast to CG metals, the grain shape and the specimen orientation can play a very important role. Often there is only a fine line between brittle and ductile behaviour and this orientation dependency may be often the origin for exceptional fracture properties when only one orientation, for example the crack divider orientation, is taken into account. This orientation dependency makes a pure grain size related discussion of the relationship of fracture toughness and grain size into a very difficult task which could also be further input for theoretical work dealing with such grain size dependencies [49–51]. Despite these clear findings, there are still a number of open questions. The intercrystalline crack path observed in iron and some other bcc metals provokes a rather poor performance in the fracture toughness. The reason for this transition from trans- to intercrystalline fracture and why in certain bcc metals this transition is not found, remains unclear. The grain shape might play a very important role for the intercrystalline crack path. Preliminary studies have shown that a transformation from an elongated into an equiaxed shape could ease the problem of low fracture toughness. But here it must be considered that in industrial SPD continuous processes will be needed which automatically leads to elongated grains, for example, thinking of extreme cold rolling or wire drawing. Other strategies to improve the toughness could attempt an additional beneficial alloying of the microstructures leading to a strengthening of the grain boundaries.

In CG materials, the COD is typically on the order of the grain size and in pure metals with low inclusion density it can be in the order of millimetre. In order to obtain a good fracture toughness in UFG and NC materials, the COD should be at least 10 or better 100 times larger than the grain size. In some of the UFG materials, this seems to be possible. The reason that shifts the formation of pores to a very late stage is however unclear. In addition, the problem of measuring the initiation toughness and crack propagation resistance is not completely solved. It may be that some adaptions of the standards are necessary to obtain comparable and technical useful fracture toughness data especially with respect to UFG and NC materials with good ductility.

## Footnotes

1 Instead of *K*_IC_, *K*_JIC_ (inferred from *J*_IC_) had to be taken, because *K*_IC_ was not available from the reference.
